# Upregulation of CD4+T-Cell Derived MiR-223 in The Relapsing Phase of Multiple Sclerosis Patients

**DOI:** 10.22074/cellj.2016.4565

**Published:** 2016-08-24

**Authors:** Aref Hosseini, Kamran Ghaedi, Somayeh Tanhaei, Mazdak Ganjalikhani-Hakemi, Shohreh Teimuri, Masoud Etemadifar, Mohammad Hossein Nasr Esfahani

**Affiliations:** 1 Division of Cellular and Molecular Biology, Department of Biology, Faculty of Sciences, University of Isfahan, Isfahan, Iran; 2 Department of Cellular Biotechnology, Cell Science Research Center, Royan Institute for Biotechnology, ACECR, Isfahan, Iran; 3 Department of Immunology, Faculty of Medicine, Isfahan University of Medical Sciences, Isfahan, Iran; 4 Department of Neurology, School of Medicine, Isfahan University of Medical Sciences, Isfahan, Iran

**Keywords:** CD4+T-cell, MicroRNAs, MiR-223, Multiple Sclerosis, Th17

## Abstract

**Objective:**

MicroRNAs (miRNA) are a class of non-coding RNAs which play key roles in post-transcriptional gene regulation. Previous studies indicate that miRNAs are dysregulated in patients with multiple sclerosis (MS). Th17 and regulatory T (Treg) cells are two subsets of CD4+T-cells which have critical functions in the onset and progression of MS. The current study seeks to distinguish fluctuations in expression of CD4+T-cell derived miR-223 during the relapsing-remitting (RR) phase of MS (RR-MS), as well as the expressions of Th17 and Treg cell markers.

**Materials and Methods:**

This experimental study used real-time quantitative polymerase chain reaction (qRT-PCR) to evaluate CD4+ T cell derived miR-223 expression patterns in patients that experienced either of the RR-MS phases (n=40) compared to healthy controls (n=12), along with RNA markers for Th17 and Treg cells. We conducted flow cytometry analyses of forkhead box P3 (FOXP3) and RAR-related orphan receptor γt (RORγt) in CD4+T-cells. Putative and validated targets of miR-223 were investigated in the miRWalk and miRTarBase databases, respectively.

**Results:**

miR-223 significantly upregulated in CD4+T-cells during the relapsing phase of RR-MS compared to the remitting phase (P=0.000) and healthy individuals (P=0.036). Expression of *RORγt*, a master transcription factor of Th17, upregulated in the relapsing phase, whereas *FOXP3* upregulated in the remitting phase. Additionally, potential targets of miR-223, *STAT1, FORKHEAD BOX O (FOXO1)* and *FOXO3* were predicted by in silico studies.

**Conclusion:**

miR-223 may have a potential role in MS progression. Therefore, suppression of miR-223 can be proposed as an appropriate approach to control progression of the relapsing phase of MS.

## Introduction

Multiple sclerosis (MS) is an inflammatory autoimmune disease of the central nervous system with an unknown etiology (1,2). Development of MS depends on both genetic and environmental factors (3,4). The clinical features of MS contain variable patterns which change over time. Among the four different phases of MS, the relapsing-remitting (RR) phase is defined as a period of acute neurological dysfunction accompanied by a degree of recovery. This is the most frequent form of MS reported thus far. At the present time primary therapy for RR-MS is interferon-β (IFN-β) which reduces disease severity (5). RR-MS patients eventually develop the secondary progressive (SP) phase, characterized by symptom progression with frequent relapse courses which may not be seen in these types of MS patients. Persistence of these symptoms with constant progression leads to a severe phase termed the primary progressive (PP) phase of MS. Unfortunately there is no agreed definition for the relapsing-progression (RP) phase of MS; indeed, it is the intermediate between the relapsing phase and progressive onset (6). 

It is believed that the immune system in MS patients incorrectly views self-antigens as foreign, eliciting a response against self (7). Migration of autoreactive T cells from the blood-brain barrier (BBB) and secretion of inflammatory cytokines induces damage to myelin sheaths. Th17 cells are the main effective cells for inflammation and pathogenesis of MS due to the secretion of several cytokines such as GM-CSF and IL-17 (8-10). On the other hand, regulatory T cells (Treg), another subset of CD4^+^ T-cells, inhibits autoimmune responses by mediating immunological tolerance to self-antigens (11). Transforming growth factor-beta (TGF-β), IL-6, and IL-1 are required for Th17 differentiation (12-14), whereas IL-23 is critical for maintenance and proliferation of Th17 cells (15). RAR-related orphan receptor γt (RORγt) is the main transcription factor in charge of Th17 differentiation which is encoded by the RORC gene (16). However Treg cells require *TGF-β* and IL-2 for differentiation from naïve CD4^+^ T-cells (17). These cells are specified by forkhead box P3 (FOXP3) (18). 

MicroRNAs (miRNA) are a new class of endogenous, noncoding RNAs which regulate expression of most genes in animals and plants (19,20). Almost every facet of cellular activity such as differentiation, metabolism and apoptosis is affected by miRNAs (21,22). We have demonstrated that immune system disorders are often accompanied by dysregulation of miRNAs (21,23). For instance, miR-155 suppresses JARID2, a DNA binding protein which leads to activation of cytokine gene expression in Th17 (24). 

Dysregulation of miR-223 in autoimmune diseases such as MS, rheumatoid arthritis (RA) and Crohn’s disease (CD) makes it a valuable diagnostic marker. miR-223 is upregulated in T lymphocytes of RA patients (25), hence this miRNA is proposed to be a biomarker for diagnosis of early stage RA patients (26). Junker et al. (27) have reported upregulation of miR223 in active MS lesions. Also, upregulation of miR-223 in peripheral blood mononuclear cells (PBMCs) and Treg cells was reported (28,29). In contrast, downregulation of this miRNA has been reported in the serum of MS patients (30). To clarify the exact role of miR-223 in MS, we carried out this study to distinguish fluctuations in expression of CD4^+T-cellderivedmiR-^223 in RR-MS patients. The association of key transcription factors involved in development of Treg and Th17 cells with transcript levels of miR-223 was also considered. We used bioinformatics tools to reveal connotation of this miRNA in pathways of Th17 and Treg differentiation. 

## Materials and Methods

### Subjects

The Institutional Review Board of Royan Institute approved the study protocol and informed consent form (Project Id. No. 91000618). All study participants provided written consent for participation. In this experimental study, 40 patients diagnosed with MS (age range: 19 to 46 years) were evaluated according to McDonald criteria (31) by a neurologist at the MS Clinic of Al-Zahra Hospital, affiliated with Isfahan University of Medical Sciences, Isfahan, Iran. From these, there were 20 patients in the relapsing phase and 20 in the remitting phase of MS. Following provision of informed consent, each patient provided 10 mL of blood, which was collected in tubes that contained Ethylenediamine-tetraacetic acid (EDTA). For patients in the relapsing phase, blood samples were taken before prescribing immunomodulatory medicine. However, remitting phase patients had previously consumed CinnoVex (IFN-β). Patients in the remitting phase provided blood samples prior to receiving the next dose of medicine. A total of 12 blood samples were collected from age and sex matched healthy control individuals who had no evidence of any allergies or infections. 

### Cell separation and RNA extraction

The obtained blood samples were immediately placed on ice and transferred to the laboratory for analysis. We used a two-step process to separate the CD4^+^ T-cells from the whole blood. In the first step, PBMCs were isolated by density gradient lymphoprep (STEMCELL Technologies, USA) according to the manufacturer’s instructions. In the second step, CD4^+^T-cells were isolated on PBMC by magneticactivated cell sorting (MACS) with a CD4^+T-^cell isolation Kit II human (Miltenyi Biotec, Germany) based on the manufacturer’s protocol. This kit is an indirect magnetic labeling system for the isolation of untouched CD4^+T^helper cells from human PBMC by elimination of cells that contain CD8, CD14, CD16, CD19, CD36, CD56, CD123, TCRγ/δ, and glycophorin A with a purity of greater than 95%. Total RNA was extracted with the TRizol® reagent (Invitrogen, USA) from CD4^+^ T-cells according to the manufacturer’s protocol. The quantity and quality of extracted RNA were verified according to the ratio of absorbance at a 260/280 nm as measured by a NanoDrop spectrophotometer (Nanodrop 1000, Thermo Scientific, USA), and by electrophoresis on a 1% agarose gel. Total RNA samples were treated with RNA-free DNase (Fermentas, Ukraine) in order to eliminate trace amounts of contaminated DNA prior to real-time quantitative polymerase chain reaction (qRT-PCR) analysis. 

### cDNA synthesis and quantitative real-time polymerase chain reaction

Total RNA samples were divided into two parts, one batch for mRNA expression analysis of key factors in Treg and Th17 cell development and the second for the miRNA expression assay. A universal cDNA synthesis kit (Exiqon, Denmark) was used for cDNA synthesis of miR-223, with *RNU48* as the reference gene (32) through a poly A tailing manner based on the manufacturer’s leaflet. Pre-designed specific primers of miR-223 and *RNU48* for qRTPCR were supplied by Pars Genome Company (Tehran, Iran). An ABI PRISM 7500 instrument (Applied Biosystems, USA) was used for the qRT-PCR analysis. All reactions were performed in triplicate using standard protocols. CDNA synthesis of key factors *TGF-β, INTERLEUKIN 23R (IL23R)* and *IL17a* was performed with a RevertAid First Strand cDNA synthesis Kit (Thermo Scientific, USA) according to the manufacturer’s protocol. The expression level of each gene was normalized vs. *18srRNA* in the same sample. All measurements were performed for three independent replicates. 

### Electrophoresis and T/A cloning

The specificity of the miR-223 primers were assessed by electrophoresis of real time PCR products on a 12% poly acrylamide gel. For exact sequence matching of miR-223 to our product, the resultant electrophoresis bands were T/A cloned into a pTZ57R/T vector (Thermo Scientific) and sent for sequencing. 

### Flow cytometry

MACS-isolated CD4^+^ T-cells were evaluated for RORγt in Th-17 cells and FOXP3 expression in Treg cells. Briefly, the isolated CD4^+^ T-cells were fixed in 4% paraformaldehyde in phosphate buffer saline (PBS) for 20 minutes at room temperature, after which they were permeabilized in 0.2% Triton X-100 for 15 minutes. Then, samples were resuspended in PBS that contained bovine serum albumin (BSA, 5 mg/mL) and subsequently stained by mouse anti-human FOXP3-PE, rat anti-human ROR γ(t)-PE, and mouse anti-human CD4-Alexa Fluor® 488 against the isotype controls (all antibodies were purchased from eBioscience, USA). All experiments were run on a FACSCalibur flow cytometer (BD Biosciences, USA) and analyzed by BD CellQuest Pro software (version 0.3). Green fluorescence was detected in a fluorescence detector 1 (FL-1). Red fluorescence was detected in FL-2.

### Statistical analysis

We used the Statistical Program for Social Sciences (SPSS) software (version 18) for all statistical tests. One-way ANOVA was utilized for comparison groups. A P<0.05 was considered statistically significant for all experiments. In order to identify validated and predicted targets of miR-223, we searched the miRTarBase (33) and miRWalk (34) databases, respectively. 

Integrative prediction analysis of ten databases by different algorithms was provided in the miRWalk database. The RNA hybrid database (35) was utilized to determine the interaction between miRNA and target mRNA. For elimination of target mRNAs which were not present in CD4^+^ T-cells, the presence of miR-223 targets in the thymus and lymph nodes was validated by the Unigene database (http://www.ncbi.nlm.nih.gov/unigene/). We used CircuitsDB (36) to explore transcription factors which could affect the miR-223 gene. Databases for annotation, visualization and integrated discovery (DAVID) (37) were implemented to reveal the most applicable pathways and molecular networks with the miR-223 targetome which were effective in Th17 and Treg differentiation. 

## Results

### Upregulation of miR-223 in CD4 + T cells in the relapsing phase of relapsing-remitting multiple sclerosis

**Fig.1:**
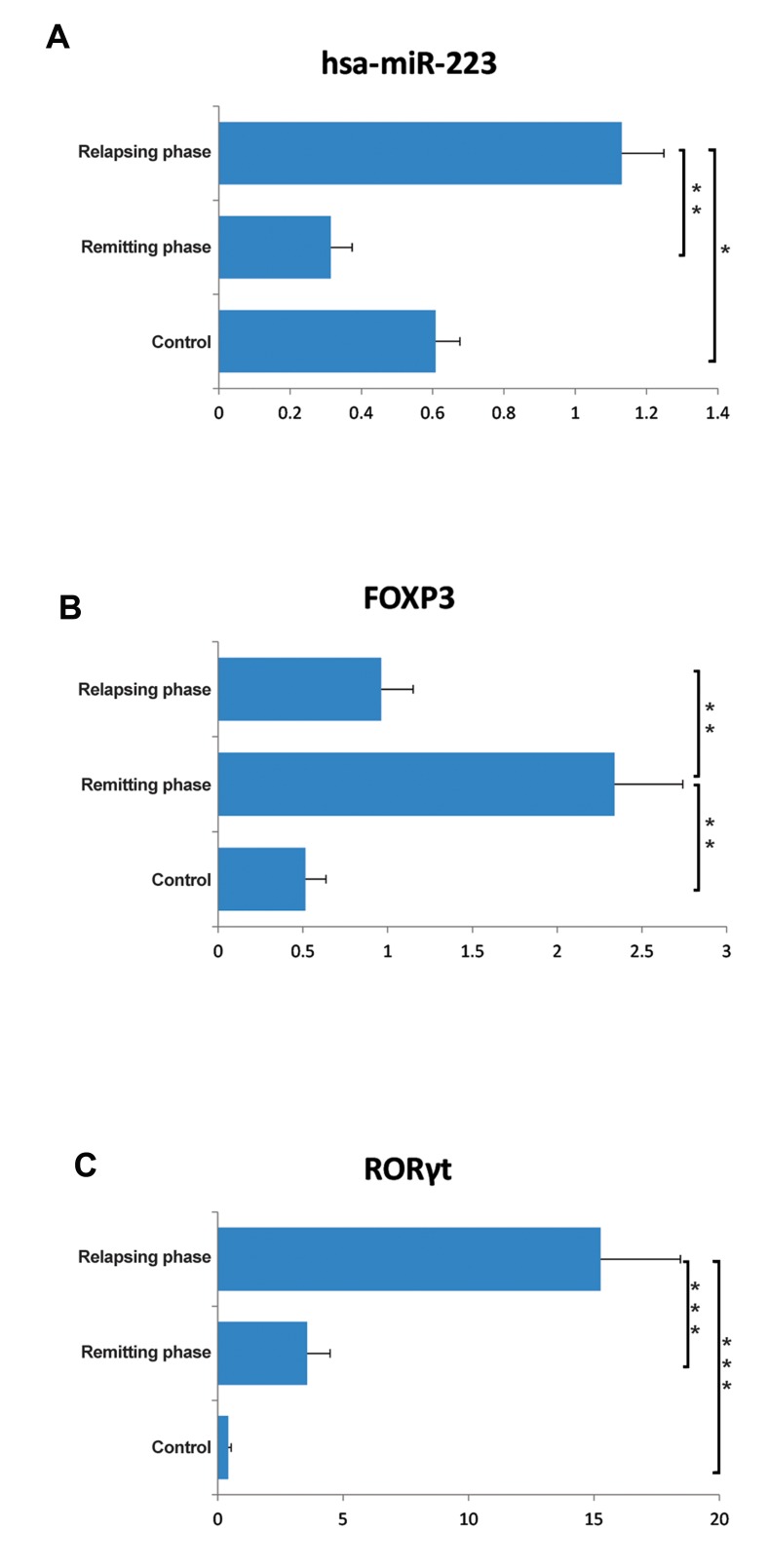
Expression of miR-223 and the percentage of Th17 and regulatory T (Treg) cells in CD4^+^T-cells. A. miR-223 significantly upregulated in the relapsing phase of relapsing-remitting multiple sclerosis (RR-MS). There was no significant difference between the remitting phase and healthy subjects, B and C. Flow cytometry results revealed an increased percentage of Th17 cells [RAR-related Orphan Receptor γt (RORγt)] in the relapsing phase. In the remitting phase, there was an elevated percentage of Treg cells [FORKHEAD BOX P3 (FOXP3)]. *; P<0.05, **; P<0.01, and ***; P<0.005, non-parametric Mann-Whitney t test.

MiR-223 expression levels were measured in subjects’ CD4^+^T-cells. We observed significant upregulation of miRNA in the relapsing phase of RR-MS compared to the remitting (P=0.000) phase and healthy controls (P=0.036). In contrast, miR-223 showed a nonsignificant downregulation in the remitting phase compared to the controls (P=0.071, Fig.1A). 

### RAR-related orphan receptor γt^+^ CD4^+^ T-cells elevated in the relapsing phase, whereas forkhead box P3^+^ CD4^+^ T-cells increased in the remitting phase of relapsing-remitting multiple sclerosis

According to flow cytometry results, the percentage of CD4^+^ T cells that contained RORγt as a key transcription factor of Th17 in the relapsing group significantly elevated compared to the remitting (P=0.0002) and control (P=0.0003) groups. CD4^+^ T-cells that carried FOXP3 as the main transcription factor for Treg cells significantly increased in the remitting phase compared to the relapsing (P=0.003) and control (P=0.001) groups (Fig.1B, C). All samples had a 92-97% purity for the CD4+ marker (data not shown).

### Dysregulation of Il-17a, *Il-23R* and *TGF-β* at the RNA level in CD4^+^ T-cells of relapsing-remitting multiple sclerosis patients 

**Fig.2:**
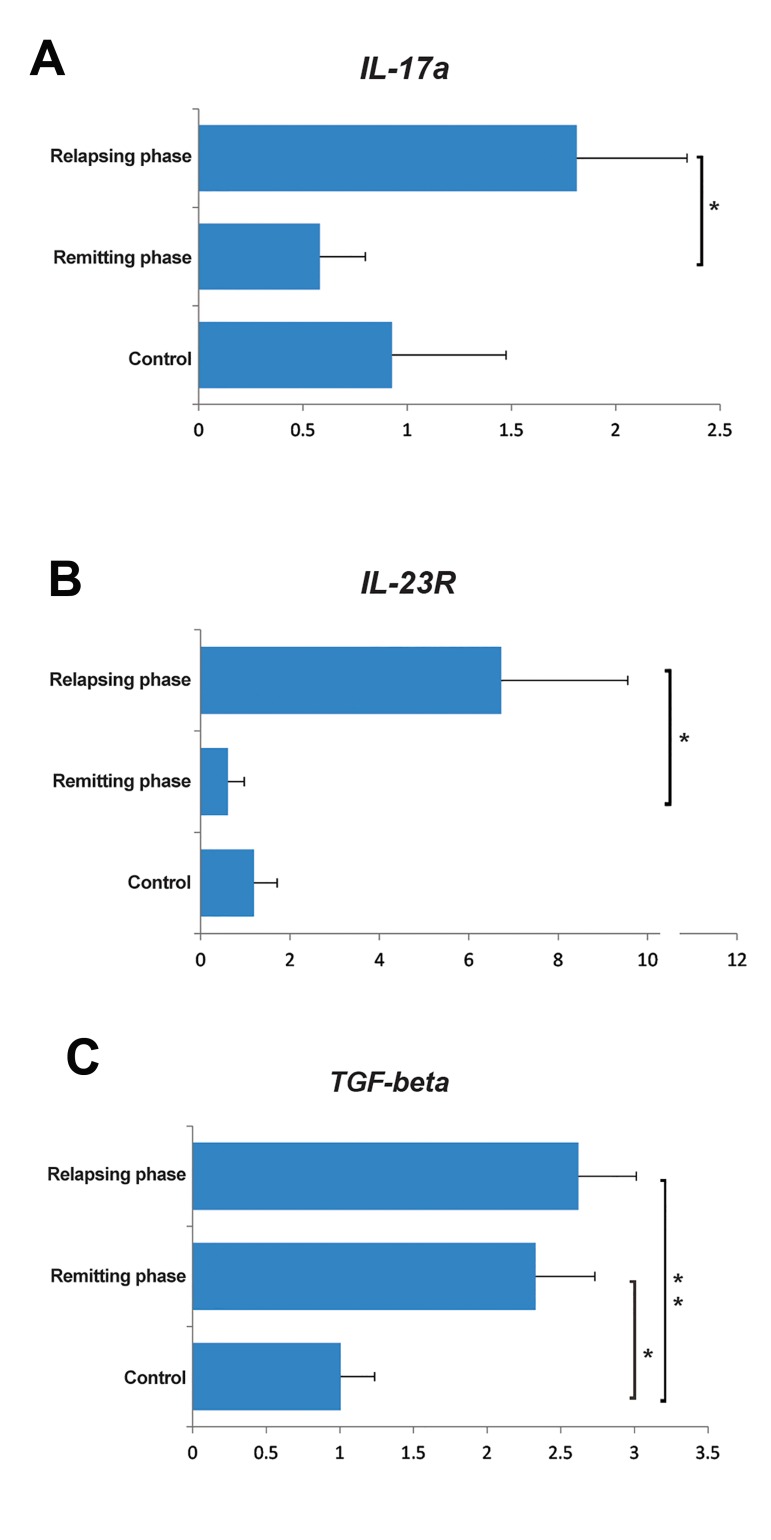
Relative expression levels of A. Interleukin 17a (IL-17a), B. *Il-23R* which represent Th17, and C. *TGF-β* activity that could positively affect both Th17 and regulatory T (Treg) cells. *; P<0.05 and **; P<0.01, non-parametric Mann-Whitney t test.

We used qRT-PCR to evaluate the expression levels of *TGF-β* and *Il-23R*, as main factors that participate in Th17 and/or Treg pathogenicity. We also evaluated Il-17a, as a Th17 cytokine marker. There was a significantly elevated transcript level of *TGF-β* in the relapsing (P=0.008) and remitting (P=0.029) groups compared to the control group. However, the difference between the relapsing and remitting groups was not significant (P=0.573). Expression of *Il-23R* increased in the relapsing phase versus the remitting phase (P=0.042), however this value was not significant between the relapsing and control (P=0.058) groups and between the remitting and control groups (P=0.815). Il-17a RNA levels significantly elevated in the relapsing compared to the remitting (P=0.023) group, however differences between the relapsing phase and control (P=0.329) groups, as well as between the remitting phase and control group (P=0.694, Fig.2) were not significant. 

### Molecular signaling pathway enrichment investigation of miR-223 targetome proposed pathogenesis of miR-223 through induction of Th17 and repression of regulatory T cells

**Fig.3:**
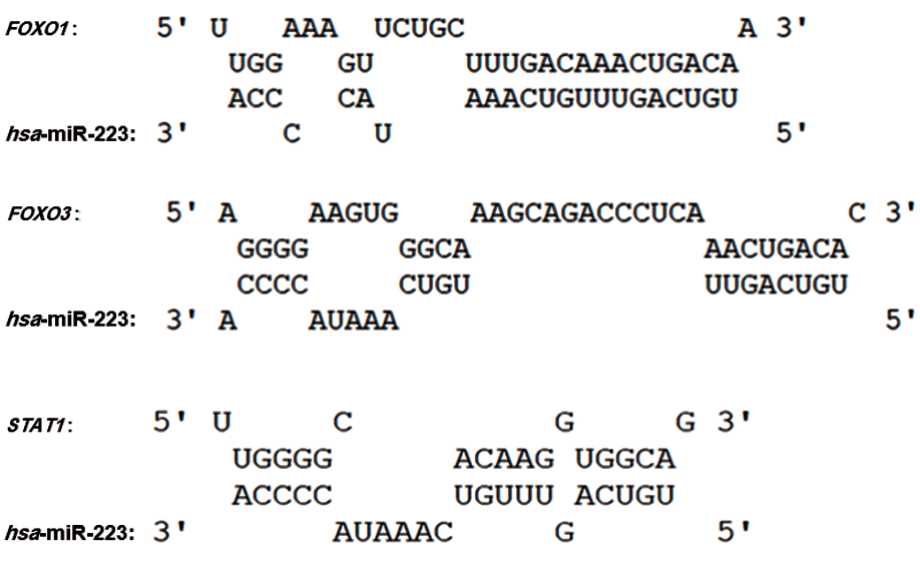
Predicted target sites for miR-223. Potential target site at the 3´-UTR of the FORKHEAD BOX O1 *(FOXO1), FOXO3* and *STAT1* genes.

**Fig.4:**
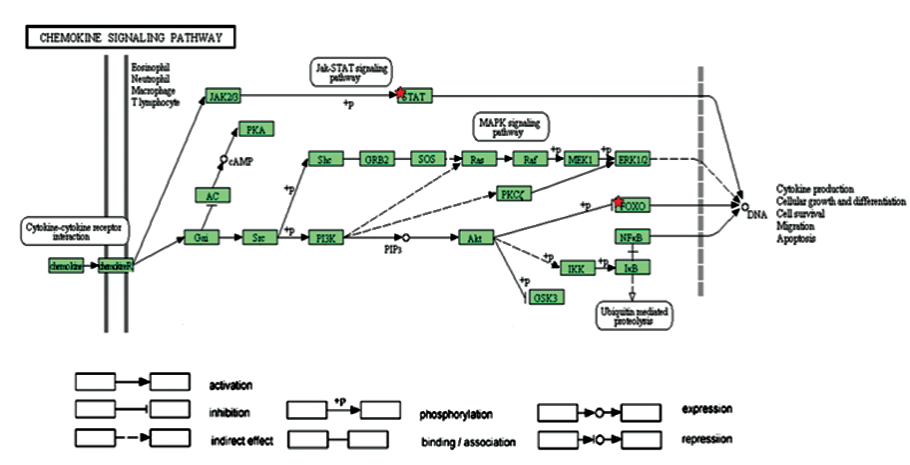
Chemokine signaling pathway. Red stars indicate predicted and validated targets of miR-223 in this pathway. STAT1 and FORKHEAD BOX O (FOXO) proteins by induction of Th1 and regulatory T (Treg) cells, respectively, could inhibit Th17 differentiation.

**Fig.5:**
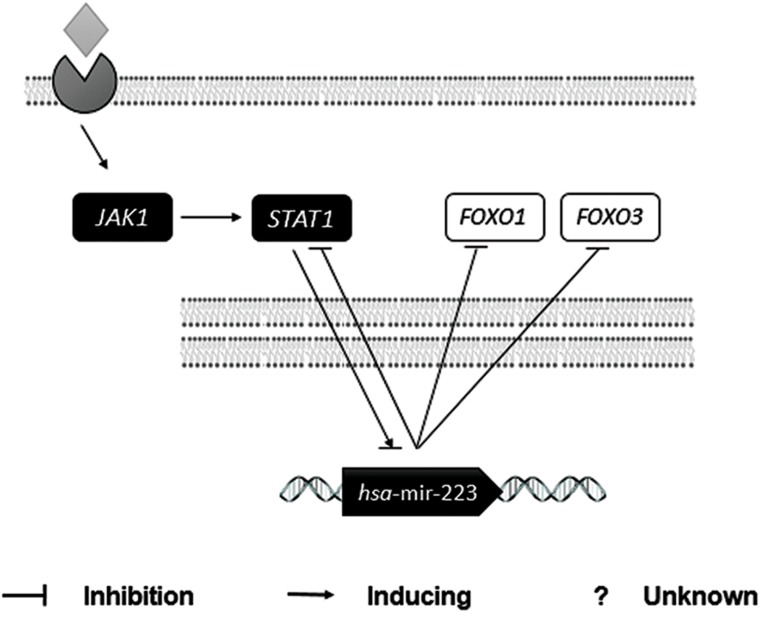
Schematic interactions of miR-223. Based on all databases used in this study, miR-223 was regulated by interferon-beta (IFN-β) by means of JAK1 and STAT1. miR-223 could regulate STAT1, forkhead box O1 (FOXO1) and FOXO3 as its targets.

**Fig.6:**
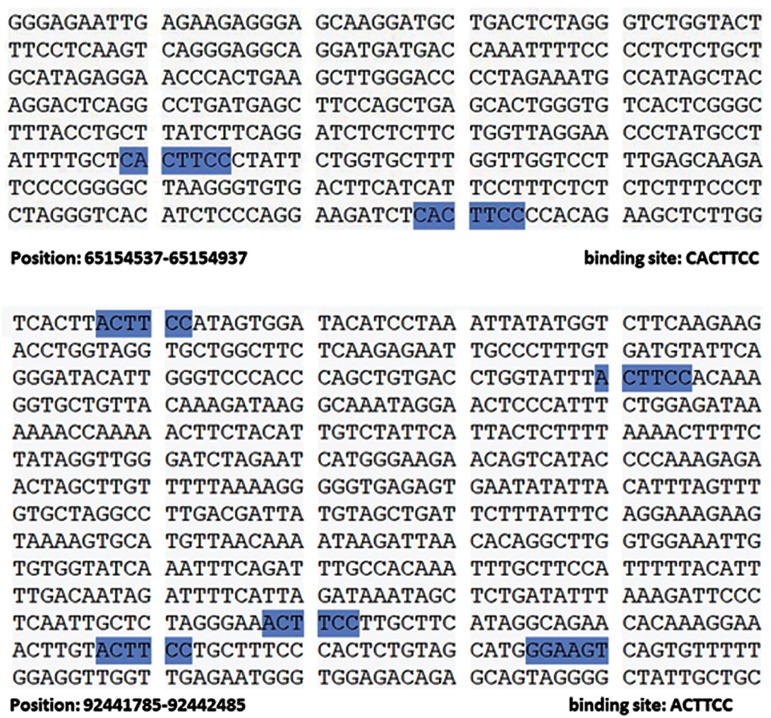
STAT1 binding sites on the hsa-mir-223 promoter sequence. A. STAT1 has two binding sites on the mir-223 promoter which bind to the CACTTCC sequence in humans and B. In mice, this transcription factor has five binding sites by a different sequence, ACTTCC. These data are based on CircuitsDB.

We performed molecular signaling pathway enrichment analysis to identify the potential role of miR-223 in MS pathogenicity by affecting Th17 and Treg differentiation. Based on miRWalk and miRTarBase databases, we identified 339 predicted and 24 validated target mRNAs (Table 1). From all predicted mRNAs determined by integrative prediction analysis in the miRWalk database, we chose those mRNAs which had been approved by at least 6 prediction databases. By using the UniGene database, we determined that 18 of the validated target mRNAs and 198 of the predicted target mRNAs expressed in the thymus and lymph nodes. Forkhead box O *(FOXO1), FOXO3* and *STAT1* mRNAs were considered as three mRNA targets of miR-223 that had vital functions for Th17 and Treg differentiation. Possible interactions between miR-223 and *FOXO1, FOXO3* and *STAT1* based on the RNAhybrid database are depicted in Figure 3. Based on CircuitsDB, 11 transcription factors related to the miR-223 gene promoter were identified (Table 2). We imputed the miR-223 targets that expressed in the lymph nodes and thymus as well as participated in Th17 and Treg differentiation into the functional annotation tool of DAVID. We elucidated a chemokine signaling pathway as an effector pathway in Th17 and Treg differentiation which could be modulated by miR-223 (Fig.4). Furthermore, our computational data have predicted two binding sites on the miR-223 promoter for STAT1 (Fig.5). As illustrated, STAT1 has two binding sites on the hsa-mir-223 promoter while, in mice this transcription factor has five binding sites in the respective promoter (Fig.6). 

**Table 1:** Predicted and validated targets of miR-223


Predicted target of miR-223									

* MAP1B*	* FGFR2*	* SLC8A1*	* KHDRBS1*	* RASSF6*	* GPM6B*	* NPY1R*	* CYP26B1*	* CLDN8*	* SLC9A2*
* NFIA*	* FOXO3*	* STIM1*	* MAP3K2*	* SGMS2*	* CCNT2*	* NRF1*	* DHX33*	* XPR1*	* SLC20A1*
* FBXW7*	* NUP210*	* TBX15*	* ARPP-19*	* E2F1*	* ANKRD52*	* ATP2B1*	* KIAA1161*	* DHRS3*	* SNCA*
* PTBP2*	* EBF3*	* TSPAN7*	* CEACAM7*	* ECT2*	* CCDC95*	* ROR2*	* AARS2*	* ZBTB47*	* SOX9*
* RASA1*	* SETBP1*	* UBE2N*	* TMED10*	* UBR1*	* C13orf15*	* C2orf64*	* WDR35*	* ORAI3*	* SP3*
* SCN3A*	* KIAA1279*	* YWHAG*	* LDB3*	* LIPH*	* GTPBP8*	* PAFAH1B1*	* ARRDC3*	* COPS2*	* SP3*
* HSP90B1*	* SACS*	* DERL1*	* AKAP10*	* SLC25A43*	* OLA1*	* ST8SIA3*	* KLHL14*	* KL*	* STAT1*
* DUSP10*	* C11orf77*	* C10orf97*	* RWDD2A*	* ZCCHC5*	* LRP12*	* PDZD11*	* FNIP2*	* QKI*	* TRPS1*
* PDS5B*	* TRIB2*	* C13orf18*	* KLF12*	* EPHA3*	* ANXA6*	* ARMCX1*	* DIP2B*	* AKAP6*	* USF1*
* ANKRD17*	* HHEX*	* RNF34*	* ADCY7*	* FAM13C1*	* ICAM1*	* HOOK1*	* ZBTB4*	* SLC4A7*	* ADIPOR2*
* FBXO8*	* HLF*	* TMEM49*	* NLRP3*	* RSBN1L*	* IFNAR1*	* SNX7*	* ZBTB26*	* SPTLC2*	* C4orf31*
* STK39*	* IL6ST*	* TBC1D10A*	* SLC26A7*	* RRAS2*	* RBPJ*	* PDE3B*	* SLC4A5*	* BAG2*	* NARG2*
* RILPL1*	* KPNA1*	* SLC37A3*	* SLC15A4*	* CLSTN1*	* RBPJ*	* C22orf28*	* PURA*	* TSGA14*	* TBC1D17*
* LMO2*	* RHOB*	* BRMS1L*	* GTSF1*	* KIFAP3*	* INHBB*	* PTPLAD1*	* ABCD4*	* ENTPD5*	* MYST3*
* CRIM1*	* LAMB1*	* MAEL*	* FOXN4*	* FOXO1*	* INPP5B*	* CTDSPL2*	* SELK*	* GREB1*	* NAT13*
* PCTK2*	* MYH10*	* ABHD13*	* CNP*	* EPB41L3*	* ITPR3*	* CMPK1*	* RAP2A*	* TRAM2*	* ADAM33*
* RPS6KB1*	* MYO5B*	* BAZ1B*	* PIGU*	* SEPT6*	* GALNTL4*	* ABCB1*	* DPF2*	* ULK2*	* GAN*
* CBFB*	* SLC11A2*	* MAFB*	* ZPLD1*	* MESDC2*	* KPNA3*	* PITPNA*	* RGS2*	* KIAA0226*	* CLPB*
* ACVR2A*	* PAX9*	* TCERG1*	* C20orf160*	* PHLPP*	* RND3*	* NUP54*	* RNF4*	* KIAA0256*	* SBF2*
* RAB10*	* LOC51035*	* RBM16*	* SFRS12*	* NUP160*	* LBR*	* PLXNB1*	* GUF1*	* SART3*	* SMC1A*
* RER1*	* PHF20L1*	* FAM5C*	* MPP7*	* SR140*	* MED11*	* ATP7A*	* BRUNOL5*	* TOX*	* SNN*
* AP1GBP1*	* PKNOX1*	* CALML4*	* CSF2*	* HEY2*	* CAPRIN1*	* POLE3*	* ATXN2*	* ProSAPiP1*	* UTP15*
* LIN54*	* ARMC1*	* DNM1L*	* CWF19L2*	* ABCA4*	* MBNL1*	* PPARA*	* SCN2A*	* MTSS1*	* KIAA1853*
* MTPN*	* CDKN2AIP*	* CDH9*	* FAM81A*	* EML2*	* MBNL1*	* FAM29A*	* SRR*	* TRIM14*	* TMTC4*
* CREB1*	* CENPN*	* CDH11*	* TTBK2*	* CNOT6L*	* ME1*	* ELOVL2*	* SLC39A8*	* MFAP3L*	* PKP4*
* PARP1*	* ANKH*	* TSPAN5*	* ZNF417*	* RNF144B*	* MEF2C*	* ULK4*	* ZFYVE20*	* TOMM70A*	* PEX3*
* XRRA1*	* NLGN2*	* CDH12*	* CTNNA2*	* WDR40A*	* ARVCF*	* RALGPS2*	* ARL6IP2*	* SRGAP3*	* SLC4A4*
* CTSL2*	* KIAA1468*	* RASGRP1*	* SEPT10*	* ZZZ3*	* MKLN1*	* USP40*	* PAPD5*	* RABGAP1L*	* CBLB*
* RNF145*	* BAI3*	* CDKN1B*	* CYB5A*	* ATRNL1*	* MMP16*	* OGFOD1*	* DKFZP686E2158*	* RP13102H20.1*	* ADAM9*
* EFNA1*	* PURB*	* TUBA1B*	* DAG1*	* SLITRK5*	* MPZ*	* PGM2*	* KLHL25*	* FAT*	* DPM2*
* ALCAM*	* RALA*	* ZNF238*	* MGC24039*	* PKD2L2*	* MSR1*	* RBM22*	* MRPS25*	* CPEB3*	* VNN1*
* F3*	* SCN1A*	* CEACAM5*	* CLEC14A*	* BRPF3*	* MX1*	* POLR3E*	* SIAH1*	* NFIB*	* C3orf15*
* F9*	* PRDM1*	* NEBL*	* C15orf26*	* EIF2C2*	* ZFHX3*	* CEP72*	* SPATA20*	* SLC17A7*	* KBTBD6*
* ACSL3*	* IFIH1*	* SORBS1*	* SPRED1*	* GOLGA1*	* ATP1B1*	* MOSPD1*	* REEP1*	* RCN2*	
* Validated targets of miR-223*									
* E2F1*	* MEF2C*	* NFIA*	* Lpin2*	* IGF1R*	* EPB41L3*	* FOXO1*	* SCARB1*	* SMARCD1*	* IRS1*
* RHOB*	* STMN1*	* Arid4b*	* CHUK*	* LIF*	* SLC2A4*	* HSP90B1*	* PARP1*	* ARTN*	* SP3*

**Table 2:** Transcription factors which could regulate miR-223 expression by binding to its promoter


Transcription factors

STAT5A
TEF-1
AML1
GATA
ETS
STAT1
GABP
LEF1
ICSBP
NF-Y
C/EBPBETA


## Discussion

In autoimmune diseases such as MS, the balance between Th17 and Treg cells is destroyed. However, miRNAs are critical post-transcription regulators that can modify differentiation pathways and potentially play a role in controlling the Th17/Treg balance.

Here, we studied the spatial expression of CD4^+^ T-cell derived miR-223 in the remitting and relapsing phases of RR-MS patients. Finding an association between the expressions of markers for two of the most critical cells in autoimmune diseases (Th17 and Treg) and transcript levels of miR-223 in CD4^+^ T-cells would expand our knowledge about the development of MS. We used bioinformatics methods to evaluate a possible mechanism where miR-223 affected Th17/Treg differentiation. Based on the predicted and validated targets of miR-223, we observed that *STAT1, FOXO1* and *FOXO3* were considered miR-223 targets in MS. Up- regulation of miR-223 in CD4^+^ T-cells from the relapsing phase of MS agreed with previous studies which showed increased expression of miR-223 in active brain lesions, PBMCs, and Treg cells of MS patients (27,38). Upregulation of this miRNA in T-cells of RA was previously reported (39). In contrast, miR-223 expression reportedly decreased in serum of MS patients (29). This discrepancy might reflect other functions for miRNA in serum and HDL (40,41). 

The main product of Th1, IFN-γ, is believed to be one of the most active suppressors for Th17 differentiation. In the absence of IFN-γ or its receptor, there will be intensified susceptibility to experimental autoimmune encephalitis (EAE) which is a common mouse model for MS (42). Binding of IFN-γ to its surface receptor will initiate the JAK/ STAT signaling cascade, leading to activation of STAT1 and to a lesser extent, STAT3 (43). Subsequently, STAT1 induces T-bet, a transcription factor which initiates Th1 lineage development (44). Activation of STAT1 causes inhibition of Th17 by means of both T-bet dependent and independent mechanisms (45). Based on miRWalk-database prediction, *STAT1* is modulated by miR-223. miR223 appears to enhance Th17 activity by suppressing *STAT1*. Interestingly, STAT1 which itself is a target of miR-223, could able to regulate the expression of this miRNA. 

As previously mentioned, the remitting group received INF-β as an immunomodulatory drug (46). IFN-β, by binding to its receptor, led to activation of JAK1, which subsequently caused phosphorylation and activation of STAT1 (47). Therefore, we speculated that STAT1 and miR-223 acted contrary to each other in terms of expression pattern and control of the Treg/Th17 balance. In agreement to our studies, Moles et al. (48) reported that in *STAT1* is a target of target of miR-223. 

Based on CircuitsDB, we have shown that there were two basic differences between humans and mice. MiR-223 has a different promoter sequence and STAT1 has different binding sites in the two species. 

Two other predicted targets of miR-223 are *FOXO1* and *FOXO3*. *FOXO1* has been validated as a target of miR-223 (49). FOXO proteins are able to bind to the FOXP3 promoter and induce Treg cells. Mice with T cell–specific deletion of both FOXO1 and FOXO3 have inadequate numbers of Treg cells (50). In contrast, inhibition of FOXO seems critical for Th17 development (51). Resistance to EAE in mice significantly increases by deletion of FOXO3 (52). We have expected that the inhibition of *FOXO1* and *FOXO3* by means of miR-223 could cause suppression of Treg cells and promote Th17 cells. 

Based on flow cytometry results, FOXP3 protein significantly overexpressed in the remitting phase compared to the relapsing and control groups. The RORγt protein significantly overexpressed in the relapsing phase compared to the other phases. 

Expression levels of *Il-23R* and *IL-17A* are associated with Th17 cells. IL-23 is required for Th17 differentiation. Therefore increased IL-17A, IL23R mRNAs in the relapsing group has resulted in increased numbers of Th17 cells. Upregulation of *TGF-β* in both the relapsing and remitting patients is intriguing. This ambiguous pattern can be explained since *TGF-β* is required for both Th17 and Treg cell differentiation (53,54). 

## Conclusion

In this study, we observed upregulation of CD4^+^T-cell derived miR-223 in the relapsing phase of RR-MS along with elevated numbers of Th17 cells and decreased Treg cells. In silico molecular enrichment analysis has shown a potential role of miR-223 in Th17 and Treg cell differentiation via the chemokine signaling pathway. However, further *in vitro* and *in vivo* experiments are needed to confirm these observations. 
